# Supplementary material for: Mean sea surface temperature changes influence ENSO-related precipitation changes in the mid-latitudes
Source: Nat Commun. 2021 Mar 5;12:1495. doi: 10.1038/s41467-021-21787-z (PMC7935986; doi:10.1038/s41467-021-21787-z)
Supplement: Supplementary file 1 — Supplementary Information [file 41467_2021_21787_MOESM1_ESM.pdf]

Supplementary material for "Mean sea surface temperature changes influence ENSO-related precipitation changes in the mid-latitudes"

Young-Min Yang[1], Jae-Heung Park[2], Soon-Il[3*] An, Bin Wang[1,4*] and Xiao Luo[4]

[1]Joint International Research Laboratory of Climate and Environment Change and Key Laboratory for Prevention of Meteorological Disaster Ministry of Education, Nanjing University of Information Science and Technology, Nanjing, 210044, China. [2]Division of Environmental Science and Engineering, Pohang University of Science and Technology, Pohang 37673, Korea. [3]Department of Atmospheric Sciences and Irreversible Climate Change Research Center, Yonsei University, Seoul 03722, Korea. 4Department of Atmospheric Sciences and International Pacific Research Center, University of Hawaii, Honolulu HI 96822, USA. email:sian@yonsei.ac.kr;bwang@hawaii.edu

Supplementary Table 1. Ocean mixed-layer heat budget. a-b, Ocean mixed-layer heat budget analysis of three different El Niño type during June-August over the equatorial central-EP (5°S–5°N, 160°W–80°W). (**a**) composite of the model with El Nino-like and (**b**) La Nina-like mean SST change.

a El Nino-like Models

Experiment	Type	$-\frac{u'\partial\overline{T}}{\partial x}$	$-\frac{\overline{u}\partial T'}{\partial x}$	$-\frac{u'\partial T'}{\partial x}$	$-\frac{w'\partial\overline{T}}{\partial z}$	$-\frac{\overline{w}\partial T'}{\partial z}$	$-\frac{w'\partial T'}{\partial z}$
**Historical**	**BW**	**0.39**	-0.07	-0.06	0.30	0.28	-0.14
	**EP**	0.04	0.01	-0.01	-0.02	**0.06**	0.01
	**CP**	**0.17**	-0.02	0.0	0.02	0.05	0.01
**RCP4.5**	**BW**	**0.35**	-0.05	-0.04	0.25	0.21	-0.11
	**EP**	0.03	0.01	-0.01	-0.01	**0.08**	0.01
	**CP**	**0.12**	-0.01	0.01	0.01	0.05	0.02
**RCP8.5**	**BW**	**0.30**	-0.04	-0.05	0.21	0.23	-0.10
	**EP**	0.04	-0.01	-0.01	-0.01	**0.08**	0.00
	**CP**	**0.10**	-0.02	0.01	0.02	0.06	0.02

b La Nina-like Models

Experiment	Type	$-\frac{u'\partial\overline{T}}{\partial x}$	$-\frac{\overline{u}\partial T'}{\partial x}$	$-\frac{u'\partial T'}{\partial x}$	$-\frac{w'\partial\overline{T}}{\partial z}$	$-\frac{\overline{w}\partial T'}{\partial z}$	$-\frac{w'\partial T'}{\partial z}$
**Historical**	**BW**	**0.28**	-0.05	-0.04	0.24	0.25	-0.11
	**EP**	0.04	0.010	-0.01	-0.01	**0.05**	0.01
	**CP**	**0.09**	-0.01	0-.01	0.01	0.02	-0.01
**RCP4.5**	**BW**	**0.31**	-0.04	-0.06	0.26	0.22	-0.10
	**EP**	0.04	0.00	-0.01	-0.01	**0.03**	0.00
	**CP**	**0.12**	-0.02	0.01	0.01	0.03	0.01
**RCP8.5**	**BW**	**0.34**	-0.06	-0.07	0.25	0.22	-0.12
	**EP**	0.02	0.00	-0.01	0.01	**0.05**	0.00
	**CP**	**0.15**	-0.04	0.01	0.01	0.06	-0.01

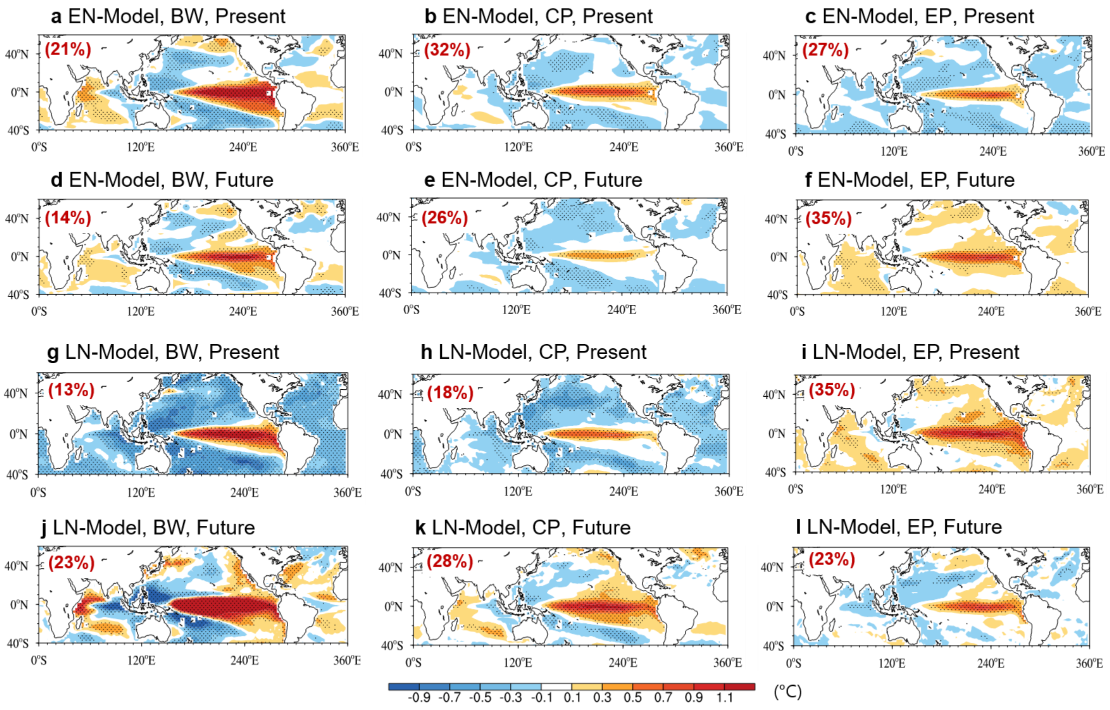

Supplementary Figure 1. Horizontal pattern of SST anomalies associated with El Niño event. a-c, Composites of October-February sea surface temperature (SST, unit: °C) anomalies for three Basin-wide (BW), Central Pacific (CP), Eastern Pacific (EP) type El Niño from the present periods of the climate models with El Niño-like mean SST change (EN-Model). The SST anomalies is averaged from October of the year before the El Niño year (−1) to February after the El Niño year (1). The "present" represents the historical run (1943–1992) from 18 climate models with two ensemble members. **d-f,** Same as **a-c** but for the "future" periods, which shows a composite of both Representative Concentration Pathway (RCP) 4.5 (2038-2-2087) and RCP8.5 (2038–2087). **g-i,** Same as **a-c** but for the models with La Niña-like mean SST change (LN-Model). **j-l,** Same as **g-i** but for the "future" periods. All data are applied to a high-pass spectral filter (13 years) to removed global warming and long-term climate variability. The stippling denotes the regions where the signal (group mean) is larger than the noise (one standard deviation of each member from the group mean). The red number in each panel shows the composite of the frequency of each El Niño type expressed as a percentage.

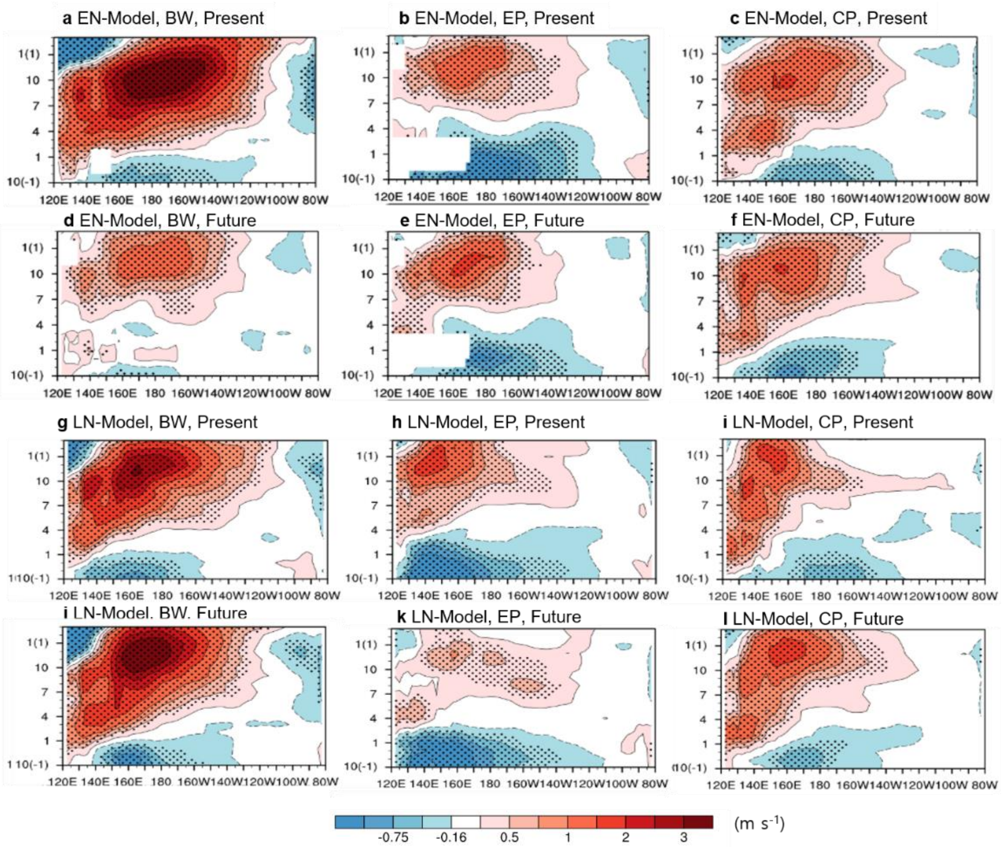

Supplementary Figure 2 Horizontal pattern of zonal surface wind associated with El Niño event. a-c, Composite of the longitude-time structure of the equatorial Pacific zonal surface wind (m s^{-1}) anomalies of Basin-wide (BW), Eastern Pacific (EP), Central Pacific (CP) type El Niño from the present periods of the climate models with El Niño-like mean sea surface temperature (SST) change (EN-Model). The "present" represents the historical run (1943–1992) from 18 climate models with two ensemble members. Each panel illustrates the composite of the zonal surface wind anomalies (in units of m s^{-1}) averaged between 5°S and 5°. The time axis starts from October of the year before the El Niño year (−1) to February after the El Niño year (1). **d-f,** Same as **a-c** but for the "future" periods, which shows a composite of both Representative Concentration Pathway (RCP) 4.5 (2038-2-2087) and RCP8.5 (2038–2087). **g-i,** Same as **a-c** but for the models with La Niña-like mean SST change (LN-Model). **j-l,** Same as **g-i** but for the "future" periods. All data are applied to a high-pass spectral filter (13 years) to removed global warming and long-term climate variability. The stippling denotes the regions where the signal (group mean) is larger than the noise (one standard deviation of each member from the group mean

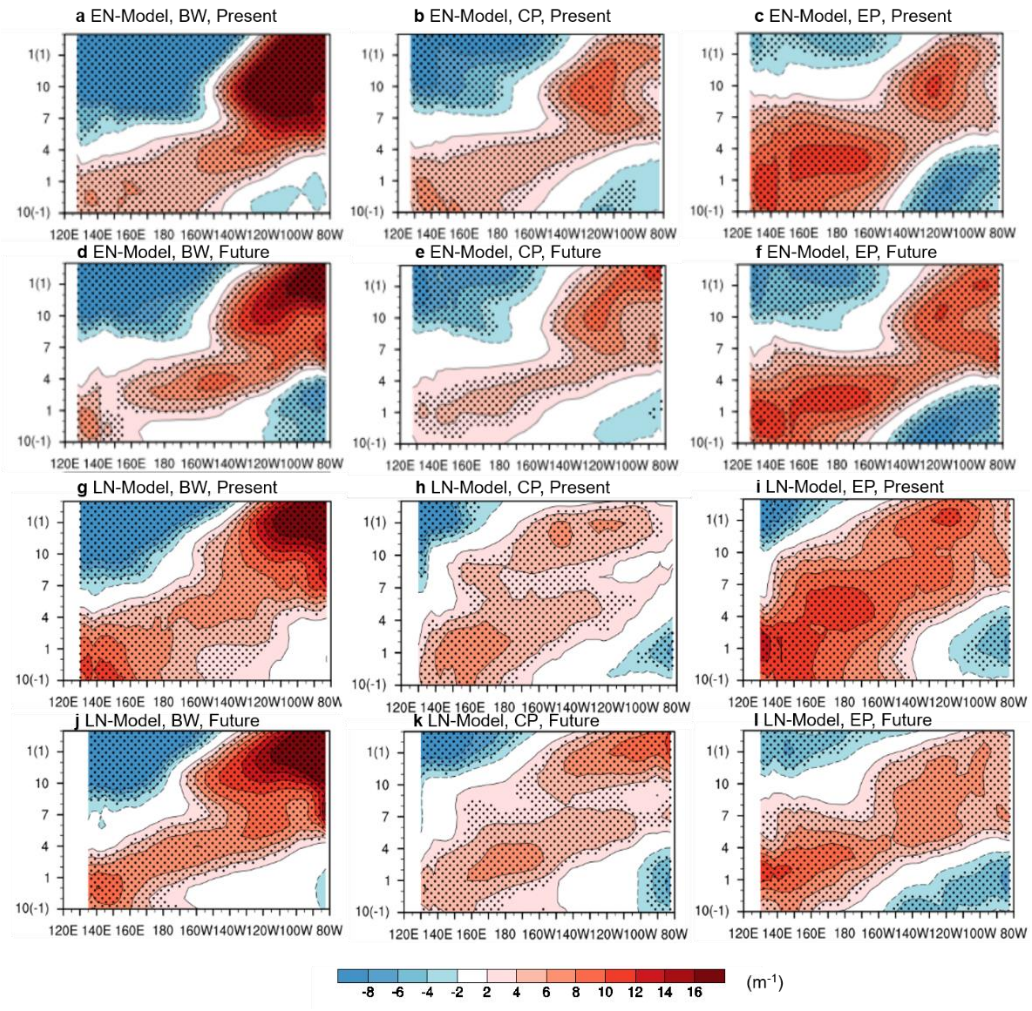

Supplementary Figure 3. Horizonal pattern of thermocline depth associated with El Niño event. a-c, Composite of the longitude-time structure of the equatorial Pacific thermocline depth (m^{-1}) at 20 °C anomalies of Basin-wide (BW), Central Pacific (CP), Eastern Pacific (EP) type El Niño from the present periods of the climate models with El Niño-like mean sea surface temperature (SST) change (EN-Model). The "present" represents the historical run (1943–1992) from 18 climate models with two ensemble members. Each panel illustrates the composite of thermocline depth (m^{-1}) at 20 °C averaged between 5°S and 5°. The time axis starts from October of the year before the El Niño year (−1) to February after the El Niño year (1). **d-f,** Same as **a-c** but for the "future" periods, which shows a composite of both Representative Concentration Pathway (RCP) 4.5 (2038-2-2087) and RCP8.5 (2038–2087). **g-i,** Same as **a-c** but for the models with La Niña-like mean SST change (LN-Model). **j-l,** Same as **g-i** but for the "future" periods. All data are applied to a high-pass spectral filter (13 years) to removed global warming and long-term climate variability. The stippling denotes the regions where the signal (group mean) is larger than the noise (one standard deviation of each member from the group mean).

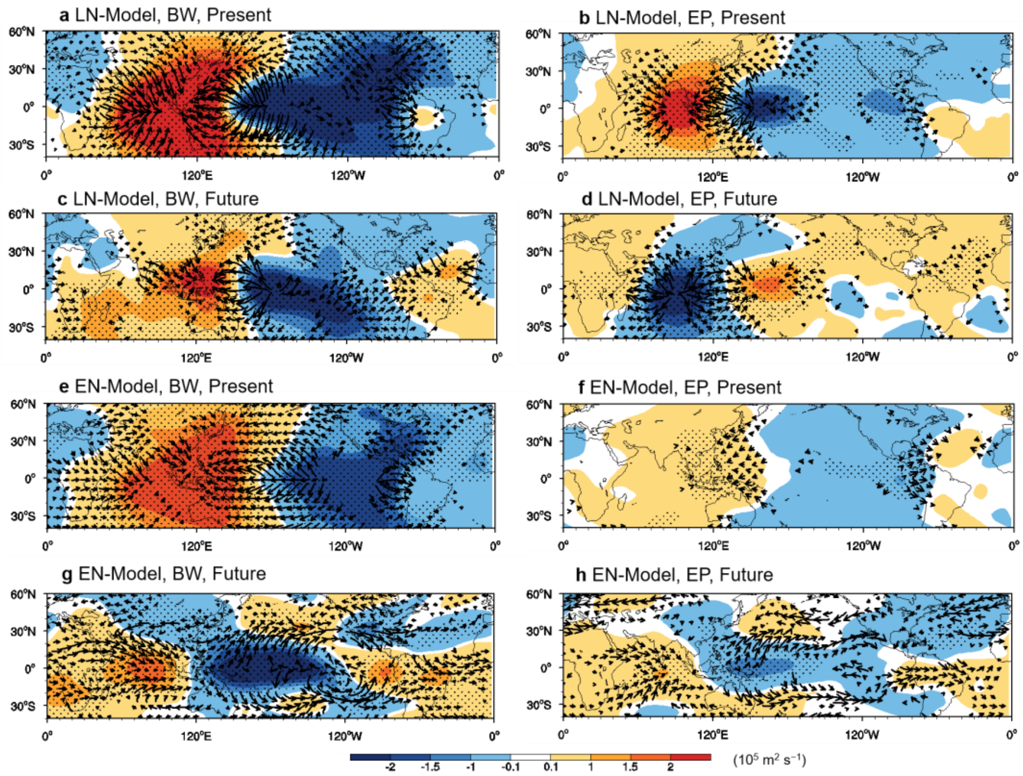

Supplementary Figure 4. Horizontal pattern of circulations associated with El Niño event. a-b, Composite of October-February velocity potential (shading, 10^5 m^2 s^{-1}) and wind anomalies (vector, m s^{-1}) at 200 hPa induced by Basin-wide (BW) and Eastern Pacific (EP) type El Niño from the present periods of the climate models with La Niña-like mean sea surface temperature (SST) change (LN-Model). The "present" represents the historical run (1943–1992) from 18 climate models with two ensemble members. Each panel illustrates the composite of depth (m^{-1}) at 20 °C averaged between 5°S and 5°. The time axis starts from October of the year before the El Niño year (−1) to February after the El Niño year (1). **c-d,** Same as **a-b** but for the difference between "present and "future" periods (future minus present). The "future" shows a composite of both Representative Concentration Pathway (RCP) 4.5 (2038-2-2087) and RCP8.5 (2038–2087). **e-f,** Same as **a-b** but for the models with El Niña-like mean SST change (EN-Model). **g-h,** Same as **e-f** but for the difference between "present and "future" periods (future minus present). All data are applied to a high-pass spectral filter (13 years) to removed global warming and long-term climate variability. The stippling denotes the regions where the signal (group mean) is larger than the noise (one standard deviation of each member from the group mean).

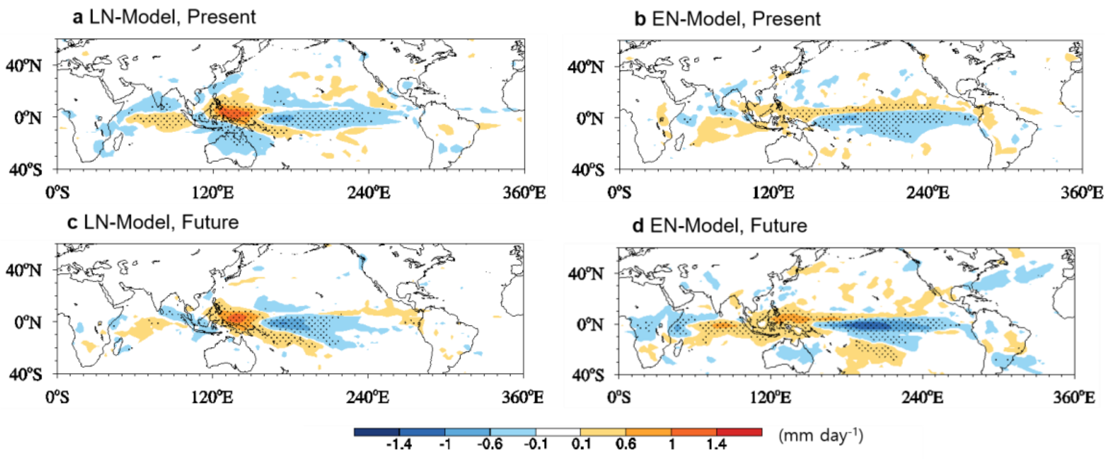

Supplementary Figure 5 Precipitation changes during neural years. a-b, Composites of boreal winter (October to February) precipitation anomalies (mm day^{-1}) during normal (or neutral) years derived from the present periods of the climate models with La Niña-like mean sea surface temperature (SST) change (LN-Model). The SST anomalies is averaged from October of the year before the El Niño year (−1) to February after the El Niño year (1). The normal years is defined if absolute values of October-February SST anomalies 5°N–5°S, 120°W–170°W over are smaller than 0.5 °C. The present represents the historical run (1943–1992) from 18 climate models with two ensemble members. **c-d,** Same as **a-b** but for the future periods, which shows a composite of both Representative Concentration Pathway (RCP) 4.5 (2038-2-2087) and RCP8.5 (2038–2087). All data are applied to a high-pass spectral filter (13 years) to removed global warming and long-term climate variability. The stippling denotes the regions where the signal (group mean) is larger than the noise (one standard deviation of each member from the group mean).

.
